# The effectiveness of a 6-week biofeedback gait retraining programme in people with knee osteoarthritis: protocol for a randomised controlled trial

**DOI:** 10.1186/s12891-023-07098-y

**Published:** 2023-12-19

**Authors:** Yi Wan, Polly McGuigan, James Bilzon, Logan Wade

**Affiliations:** 1 Department for Health, University of Bath, Bath, UK; 2 Centre for the Analysis of Motion, Entertainment Research and Applications (CAMERA), University of Bath, Bath, UK; 3 Centre for Sport Exercise and Osteoarthritis Research Versus Arthritis, University of Bath, Bath, UK

**Keywords:** Knee osteoarthritis, Knee loading, Gait retraining, Biofeedback, Biomechanics, Stair, Sit-to-stand

## Abstract

**Background:**

Gait retraining is a common therapeutic intervention that can alter gait characteristics to reduce knee loading in knee osteoarthritis populations. It can be enhanced when combined with biofeedback that provides real-time information about the users’ gait, either directly (i.e. knee moment feedback) or indirectly (i.e. gait pattern feedback). However, it is unknown which types of biofeedback are more effective at reducing knee loading, and also how the changes in gait affect pain during different activities of daily living. Therefore, this study aims to evaluate the acute (6 weeks of training) and chronic (1 month post training) effects of biofeedback based on personalised gait patterns to reduce knee loading and pain in people with knee osteoarthritis, as well as examine if more than one session of knee moment feedback is needed to optimise the gait patterns.

**Methods:**

This is a parallel group, randomised controlled trial in a symptomatic knee osteoarthritis population in which participants will be randomised into either a knee moment biofeedback group (*n* = 20), a gait pattern biofeedback group (*n* = 20) or a control group (*n* = 10). Supervised training sessions will be carried out weekly for six continuous weeks, with real-time biofeedback provided using marker-based motion capture and an instrumented treadmill. Baseline, post-intervention and 1-month follow-up assessments will be performed to measure knee loading parameters, gait pattern parameters, muscle activation, knee pain and functional ability.

**Discussion:**

This study will identify the optimal gait patterns for participants’ gait retraining and compare the effectiveness of gait pattern biofeedback to a control group in reducing knee loading and index knee pain. Additionally, this study will explore how many sessions are needed to identify the optimal gait pattern with knee moment feedback. Results will be disseminated in future peer-reviewed journal articles, conference presentations and internet media to a wide audience of clinicians, physiotherapists, researchers and individuals with knee osteoarthritis.

**Trial registration:**

This study was retrospectively registered under the International Standard Randomised Controlled Trial Number registry on 7th March 2023 (ISRCTN28045513).

## Background

Knee osteoarthritis (KOA) is a common musculoskeletal disease which can have a significant impact on physical function and quality of life, especially in older populations [1]. Low-level micro-damage to intra-articular structures can be managed and regenerated in healthy populations, however, once the cartilage begins to degenerate due to excessive or uneven loading in people with KOA, the joint responds negatively to the compressive forces by further increasing joint loading, which accelerates the progression of KOA [2]. Sub-optimal gait patterns prior to injury, or an injury-induced gait pattern, may lead to greater knee loading on the affected side and a shift in the specific loading area of the cartilage [2]. Therefore, increased knee mechanical loading has a strong correlation with the progression of the disease [3, 4], and therapeutic intervention for KOA should focus on reducing knee loading during activities of daily life.

Previous research has found that knee moments, primarily in the frontal plane, have a strong correlation to knee joint contact forces [5, 6]. Considering that the majority of knee loading is distributed on the medial compartment, knee adduction moment (KAM) has become a common surrogate for knee joint contact force during movement [7, 8]. Recent studies have demonstrated that knee flexion moment (KFM) can also be a dominant predictor of the lateral knee joint contact force in both KOA and healthy populations [9]. Therefore, reducing the magnitude of knee moments in both frontal plane and sagittal plane during daily activities should be a target in research and clinic rehabilitation.

Gait retraining has been used as an effective, non-invasive strategy to slow down the progression of KOA by reducing the magnitude of knee loading [10]. Previous studies have reported the benefits of a smaller foot progression angle (FPA) for reducing the first peak KAM in both healthy and KOA populations [11, 12]. Greater step widths have been shown to reduce both first and second peak KAMs during walking and stair climbing by reducing the frontal plane ground reaction force moment arm length [13–16]. Finally, smaller step lengths have been found to reduce the peak KAM and KAM impulse in healthy weight and obese adults and KOA population [17–19]. Due to the complexity of walking and various anatomical differences between people, reducing knee loading using a fixed FPA is unlikely to be achievable, and thus a combination of FPA, step width and step length would likely facilitate a personalised gait modification that is comfortable and effective at reducing knee loading [20].

To enhance the performance and learning effect of gait retraining, biofeedback has been employed to provide real-time body information using visual and haptic interfaces [10, 21–23]. By interacting with the live feedback through changes in body position, participants learn how to modify and control their body movement during a motor task, which is gradually internalised over time [24]. Biofeedback is generally employed in two forms; direct feedback provides information about the target outcome itself, in this case knee loading, which participants need to reduce; while indirect feedback provides information about other outcomes such as FPA, step width and step length, which could affect the desired target outcome (i.e. knee loading). Direct feedback has been found to have a better effect on skill learning and retention compared to indirect feedback [25, 26]. however, previous evidence was based on healthy participants [23, 27], and no experimental study has compared different types of feedback in parallel. In addition, while knee loading may be the most effective feedback method in research, it is currently impossible to provide this type of feedback outside a lab setting. Therefore, it is important to explore the effectiveness of indirect feedback (gait pattern) methods that are more feasible in a day-to-day clinical and home settings.

This study will primarily explore the effectiveness of gait pattern feedback to reduce knee loading and knee pain during a 6-week gait retraining programme, and knee moment feedback group will be exploratory to identify the number of sessions needed to determine the optimal gait pattern over 6 weeks. In a long-term gait retraining programme, the gait patterns identified in the first session may not be optimal due to familiarisation with the environment, skin markers and desired gait alterations, as well as any learning effect or muscle activation adaptations [28]. Therefore, this study will explore if a participant’s optimal gait pattern changes or improves over time when provided with direct knee moment feedback, and how many sessions are required to identify a stable optimised gait pattern. Furthermore, there have not been any studies exploring the translative effect of gait retraining intervention into daily activities other than walking (e.g. sit-to-stand or stair climbing). Addressing these knowledge gaps in the literature will produce quantifiable outcome measures to identify and support the implementation of gait retraining for future clinical rehabilitation in KOA populations.

The primary objective of this study is to compare the effectiveness of gait pattern feedback to a control group, during a 6-week gait retraining programme in a KOA population. The secondary objectives are as follow:

1. Explore the evolvement of gait patterns (FPA, step width and step length) when using knee moment biofeedback over time.
2. Determine the optimal number of knee moment biofeedback sessions to identify the best combination of gait patterns (FPA, step width, step length) and quantify individual differences in the timescale for adaptation.
3. Compare the similarity between gait pattern feedback and knee moment feedback to reduce KAM and pain.
4. Explore and compare the successful internalisation of these two types of biofeedback gait retraining over time, through dual tasks, muscle activation and one-month gait retention.
5. Explore the translative effect of these biofeedback gait retraining programmes on general daily activities.

## Methods

### Study design

This is a parallel group, randomised controlled trial, where participants will be randomly allocated into three groups; knee moment biofeedback group, gait pattern biofeedback group and control group. In participants with bilateral KOA, the most painful knee will be studied. The primary outcome will be KAM, while knee pain and functional ability will be considered secondary. All other outcomes will be exploratory outcome variables. The primary comparison will be between gait pattern feedback group and control group, while the comparison between gait pattern feedback group and knee moment feedback group will be exploratory.

### Recruitment and consent process

Participants will be recruited through NIHR Clinical Research Network, local hospitals, and social media. Clinical participants will be identified through routine consultations, physio classes, and patient database search. General public participants will be recruited through advertisements from the University website, social medial platforms, and by word of mouth. Interested participants will contact the research team, followed by further eligibility screening by the researcher. A participant information sheet will be emailed to eligible participants, and a written informed consent will be obtained by the researcher.

### Patient and public involvement

This study was conducted as part of our Versus Arthritis Centre for Sport Exercise and Osteoarthritis Research (CSEOR) and developed in accordance with the our Patient and Public Involvement (PPI) strategy [29]. The specific study protocol and biofeedback methods were co-developed with local patients and therapists and reviewed and endorsed by our national CSEOR PPI Advisory Panel. We will continue to collate participant feedback on patient experiences throughout the trial ensuring that we capture comments related to future improvements to overcome barriers to engagement, training delivery and adverse effects. This will help to understand and improve current and future intervention study designs.

### Study population/sample size

A sample size calculation was performed based on randomisation with stratification. Using an alpha level of 0.05, a power of 0.8, and an effect size estimates of 1.01 for KAM based on a previous gait retraining study with a similar design compared to our primary comparison (gait pattern group vs control group) [22]. Given that the comparison between gait pattern group and knee moment group is a secondary objective and no study has done the similar design, we will stick with the sample size needed for our primary objective. Seventeen participants are required for each experimental group. Thus, a total of thirty-four participants are needed to adequately power the study and detect significant differences in KAM between groups. Using a conservative estimate of twenty percent drop-out rate, the aim is to recruit twenty participants per group. For control group, we used an allocation ratio of 2:1 requiring recruitment of ten participants. This unequal allocation ratio for control group has been shown to increase the amount of data in the intervention groups where individual responses are likely more variable [30, 31]. In total, this study will recruit fifty participants.

### Eligibility criteria

Inclusion criteria:

1. Clinical diagnosis of KOA.
2. Aged between 45 and 69 years.
3. BMI ≤ 40.0 kg/m^2^.
4. Current knee pain (minimum numeric rating score 2).
5. Be able to walk without an assistive device for at least 15 consecutive minutes.

Exclusion criteria:

1. Body mass index (BMI) > 40.0 kg/m^2^.
2. Valgus knee alignment > 5° (this will be assessed in the baseline assessment session).
3. History of knee replacement or tibial osteotomy.
4. Conditions other than KOA that could affect walking (e.g. amputation, severe back pain, severe peripheral vascular or heart disease and neurological or developmental disease or lower limb surgery within the past 6 months);
5. Inability to adopt an altered gait due to previous injury or surgery.
6. Use of any orthotic equipment.
7. Corticosteroid injection or oral intake within the past 6 weeks.
8. Participation in a new structured exercise program or other treatment for KOA within the past 3 months, or planning to commence in the next 3 months.

### Randomisation

After the baseline assessment, participants will be randomly allocated (2:2:1) to the knee moment biofeedback group, gait pattern biofeedback group or control group, using a block randomisation plan by the researcher. Blinding is not achievable in this intervention study, and therefore neither participants nor researcher will be blinded.

### Experimental setting

All sessions will be conducted in the Applied Biomechanics Suite at the University of Bath. During gait retraining sessions, participants will walk on an instrumented treadmill. During assessment sessions, participants will perform overground walking, sit-to-stand and stair climbing on force platforms embedded in the floor and a portable force platform embedded on the first step of a two-step stair. During each session, a twelve camera, three-dimensional optoelectronic camera system (Arqus and Miqus, Qualisys, Sweden) will be used to collect motion capture data. Reflective markers will be placed on the distal phalanx of the first toe, metacarpal phalangeal joints 1 and 5, calcaneus, tuberosity of 5th metatarsal, sustentaculum tali of calcaneus, lateral apex of the peroneal tubercle, medial and lateral malleolus of the ankle, tibial tuberosity, medial and lateral femur epicondyles, left and right anterior superior iliac spine and posterior superior iliac spine, left and right iliac crest, vertebrae C7, suprasternal notch of the manubrium and left and right acromion process. Four marker clusters on rigid plates will be placed on the lateral side of the shank and thigh of each leg. Lower limb muscle activation data will be collected by surface electromyography (EMG), including vastus lateralis, vastus medialis, rectus femoris, biceps femoris, medial gastrocnemius, soleus, tibialis anterior, and gluteus medius.

### Outcome measures

#### Demographics

Age, sex, and previous injury/surgery history will be asked at the baseline assessment session (week 0), along with weight and height measurements.

#### Qualitative assessment

The Oxford Knee Score questionnaire will be carried out only at baseline assessment session (week 0), as a reference of the baseline severity of KOA. The Western Ontario and McMaster Universities Arthritis Index (WOMAC) will be assessed at all three assessment sessions (weeks 0, 6 and 10) to evaluate the condition of KOA, including pain, stiffness, and physical functioning of the knee joint. Numeric Rating Scale (NRS) of pain on the target knee joint will be assessed after the final trial of each activity at all assessment sessions (week 0, 6 and 10).

#### Free-living energy expenditure

All participants will be asked to wear a wrist-mounted physical activity monitor (GENEActive accelerometer) continuously for 6 weeks from the baseline assessment session (week 0 – 6). Free-living energy expenditure will be estimated using previously published algorithms [32]. Weekly activity log will be used from one week prior to the first training session till the end of the training (week 0 – 5). Participants will record the type of activity, duration, intensity/frequency and how they feel every day for 6 weeks.

#### Biomechanical outcomes

Across all groups, we will collect the biomechanical outcomes measures listed below in all three assessment sessions (weeks 0, 6 and 9). In the intervention groups, we will collect all these measures during training sessions 1 and 6, while training sessions 2–5 will only record knee loading and gait pattern data (no muscle activation data). For the control group, all these measures will only be recorded during sessions 1 and 6.

1. *Knee loading data* will be collected and calculated as KAM, KFM and KAM impulse. Hip moment will also be calculated as exploratory information.
2. *Gait patterns data* will be collected as FPA, step width, and step length. Other gait parameter such as trunk lean and pelvis tilt will also be calculated.
3. *Muscle activations data* will be measured by surface EMG from lower limb muscles using surface electrodes. Before placement, electrode locations on each participant’s affected leg will be palpated, shaved, lightly abraded, and cleaned with an alcohol swab. EMG normalisation will be performed using maximum voluntary contraction (MVC) tests for all these five muscle groups (knee extension, knee flexion, ankle plantar flexion, ankle dorsiflexion, hip abduction).

### Overview of the programme

**Fig. 1:**
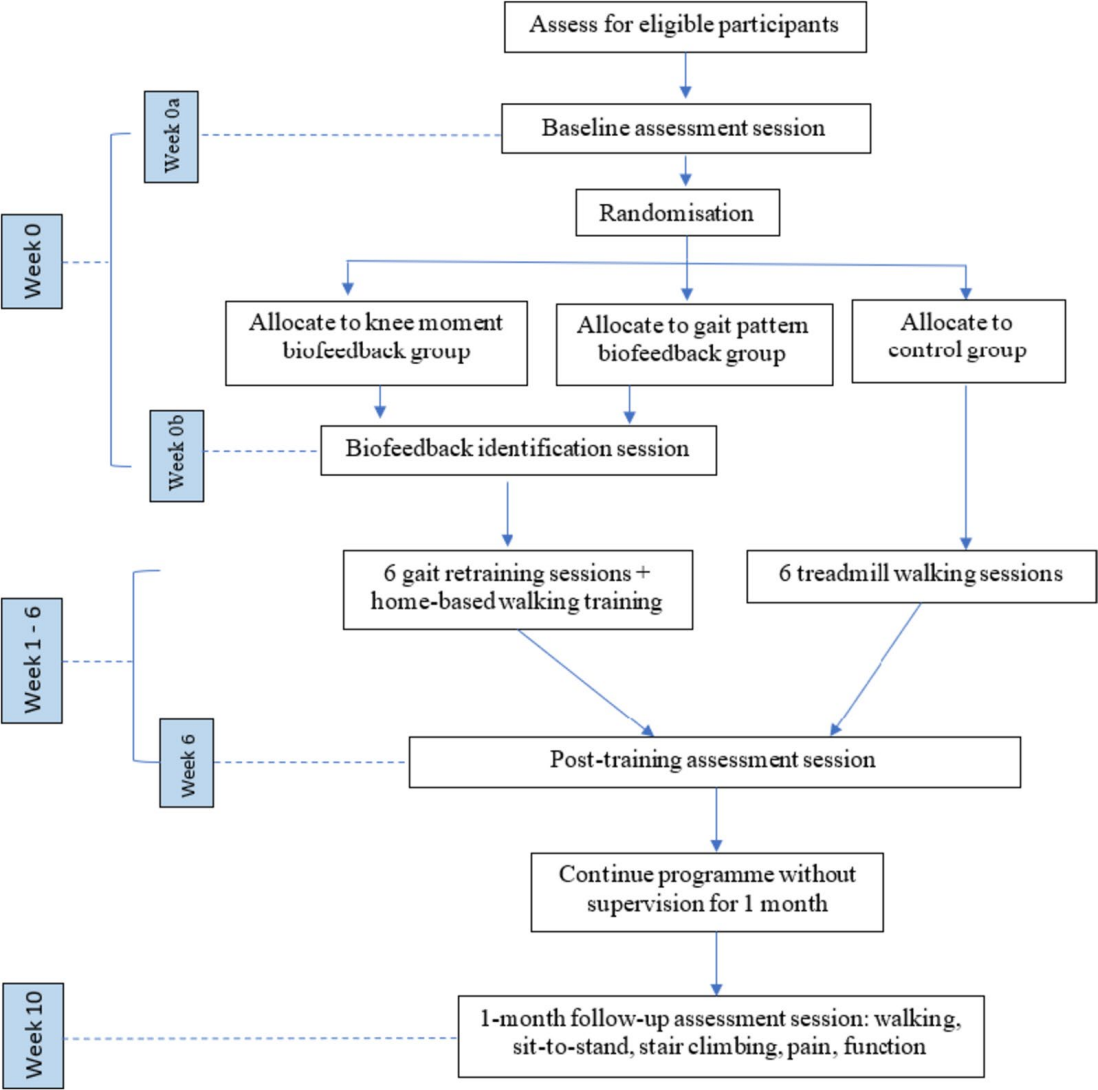
Trial flow chart

Participants in the intervention groups will attend the lab a total of nine times, while the control group will attend a total of seven times. Firstly, all participants will attend the baseline assessment session (week 0a), after which a randomisation procedure will be carried out where participants will be randomly allocated to each group. Then, participants in the intervention groups will attend a biofeedback identification session in the same week (week 0b) to identify their optimal gait patterns. From week 1 to week 6, participants will start the weekly gait training sessions. Intervention groups will have real-time biofeedback along with treadmill walking, while the control group will perform treadmill walking for the same length of time without any instruction or feedback. The last training session (week 6) will finish with the post-training assessment session. Finally, a retention assessment session will be carried out one month later (week 10) for the two intervention groups only. Between the post-training assessment session (week 6) and follow-up assessment session (week 10), there will be no training or instructions of any kind provided. Figure 1 details the trial flow chart.

### Assessment sessions

There will be three assessment sessions (Fig. 1) throughout the programme (week 0, 6 and 10). All three assessment sessions will be identical, except the Oxford Knee Score assessment will be performed in the baseline assessment session (week 0). The WOMAC questionnaire will be assessed at the beginning of each assessment session, after which, reflective markers and EMG electrodes will be attached to the participant. Overground walking, stair ascent and descent, and sit-to-stand will be performed in a block randomised order. All activities will be performed under their preferred overground gait condition, and each activity will be repeated five times. The overground walking speed during each assessment session will be controlled to be within 20% of the baseline overground walking speed. At the end of the final trial of each activity, participants will be asked to report their perceived knee joint pain using NRS.

### Biofeedback identification session

This session (Fig. 1) will only be conducted in the two intervention groups, and both intervention groups will receive knee moment feedback to identify their optimal gait patterns. Reflective markers and EMG electrodes will be attached. Participants will be instructed to walk on the instrumented treadmill and try various gait patterns (FPA, step width and step length), aiming to reduce the real-time knee moments graphs shown on the screen in front of them. They will first be instructed to try different FPAs, then try different step widths and finally different step lengths, to identify their optimal values for each gait parameter. Then, participants will be given 5 min to explore a combination of gait patterns that can consistently keep the knee moment graphs at a low level, in a symmetric, pain free and sustainable method. This 5-min period will be recorded to identify the FPA, step width and step length that most effectively reduces KAM for each individual.

### Gait training sessions

**Fig. 2:**
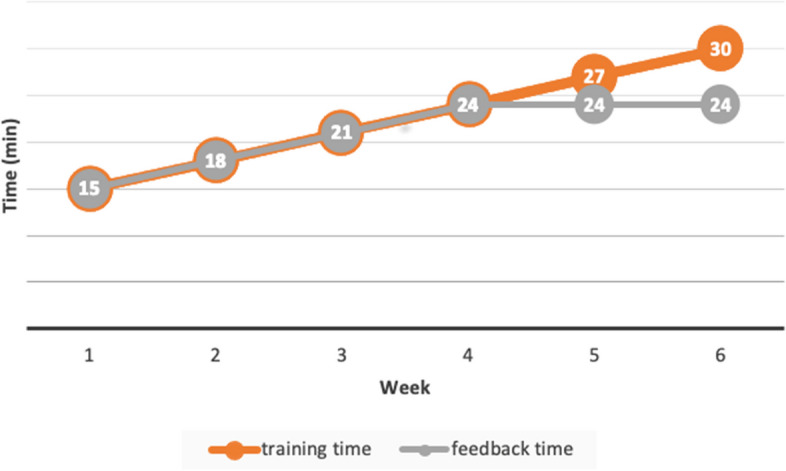
Schedule of training time and feedback time during gait retraining sessions from week 1 to week 6. The orange line represents treadmill walking time, which is identical for all three group. The grey line represents feedback time, which is identical for the two intervention groups

From week one to week six, participants will attend weekly training sessions to perform constant preferred speed walking on the treadmill. The treadmill walking speed might be different from the preferred overground walking speed during assessment sessions, considering the familiarity and energy expenditure on the treadmill. The constant treadmill walking speed will be set to be 0.1 m/s slower than their choice of preferred speed on treadmill, considering the increased difficulty while interacting with feedback during gait retraining. Training time will gradually increase from 15 min in week 1 to 30 min in week 6 (Fig. 2). For the two intervention groups, real-time biofeedback graphs will be provided with personalised targets while walking on the treadmill. The feedback will be graphical data, not numerical data. The control group will perform treadmill walking for the same length of time without any feedback or instruction. The length of feedback time in the intervention groups will go up by the same amount (three minutes) per week until week 4, while it will remain the same (24 min) from week 5 onwards (Fig. 2).

For the participants in the two intervention groups, outside of the lab they will be asked to practice the new gait pattern at least 10 min per day, while also trying to maintain this pattern during the rest of their daily walking. All participants will be given a new weekly activity log and a physical activity monitor at the end of each training session.

#### Knee moment biofeedback gait retraining group

**Fig. 3:**
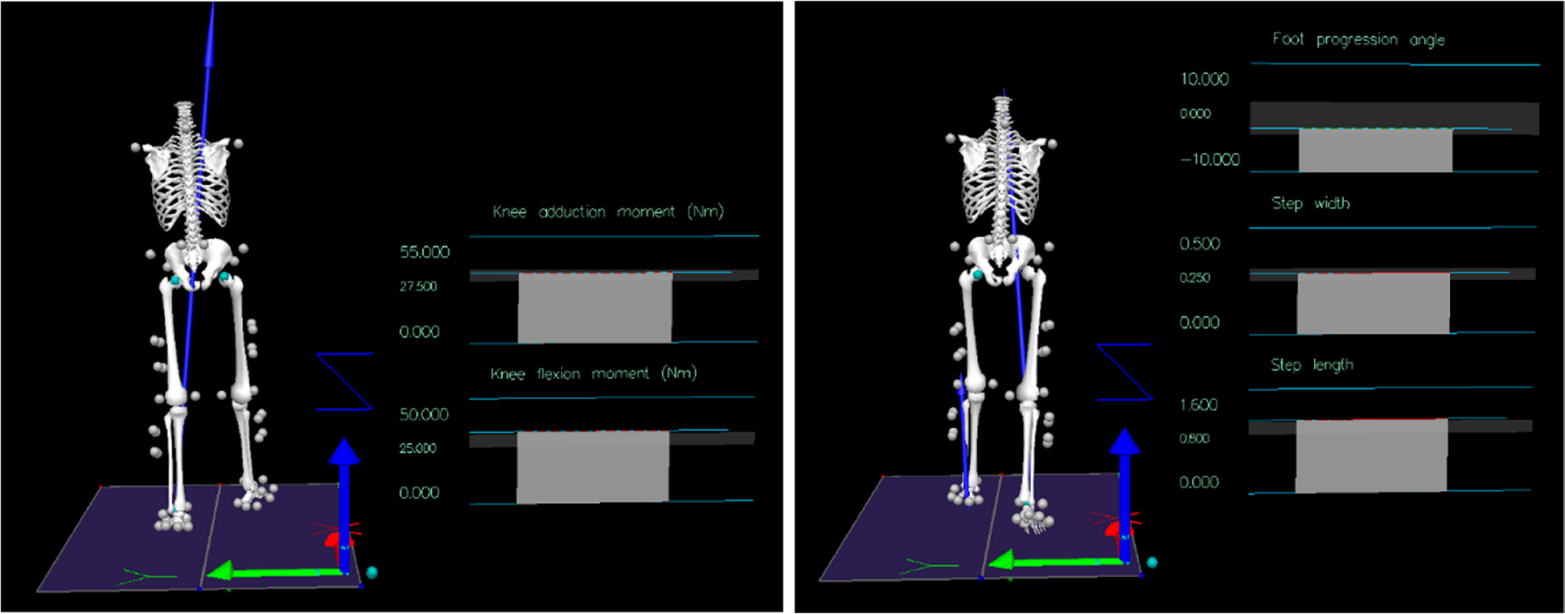
Example of real-time knee moment feedback (left) and gait pattern feedback (right). The light grey shaded area in each graph is the customised target for each parameter

Real-time feedback of their live KAM and KFM graphs along with their visual skeleton will be provided on the screen in front of the treadmill, using the Visual 3D real-time plugin (C-Motion, Inc.) (Fig. 3). During each training session, they will try to reduce the1^st^ peak KAM graph with a target of 10% reduction of their baseline level and not to increase KFM by 1% of their baseline level [33, 34], when modifying their gait patterns (FPA, step width and step length). As such, their optimised gait pattern may change over the six weeks.

#### Gait pattern biofeedback gait retraining group

Real-time feedback of their live gait patterns (FPA, step width and step length) graphs with their personalised gait pattern target and their visual skeleton will be presented on the screen in front of the treadmill through the Visual 3D real-time plugin (C-Motion, Inc.) (Fig. 3). The participants individualised gait pattern target will depend on the outcomes from the identification session (week 0b, Fig. 1), which will be the same during each training session. Participants will be told to match the target as much as possible.

#### Dual-task

At the end of each training session for the two intervention groups*,* participants will perform the dual-task (in this case Visual Stroop test), where words that describe a colour (e.g. red, blue) will display on the screen in different colour text (e.g. the word red will be display in blue text), at 2 s intervals. Participants will need to respond with the colour of the text while maintaining the modified gait pattern during treadmill walking.

### Delayed control intervention

While control participants may have a strong preference to receive the intervention, they will be offered a delayed control intervention after they finish the study (Week 6). It will involve a biofeedback identification session and six in-person biofeedback gait retraining sessions.

### Withdraw criteria

Participants will be informed that they are free to withdraw from the study prior to, during and after all data collection sessions. Once the data have been collected, access to change or remove a participant’s data will be limited, as we need to manage information in specific ways for the research to be reliable and accurate. If a participant withdraws from the study, we will keep the already obtained data.

### Statistical analysis

Study outcomes will be assessed quantitatively. The primary outcome will be KAM and KAM impulse, while NRS pain and WOMAC scores will be considered secondary. All other biomechanical outcomes (e.g. spee, EMG data, gait pattern variables) will become exploratory outcome variables in nature. The primary success of the trial will be based on a comparison of KAM reduction between week 0 and week 6, while the success of retention will be based on a comparison of between week 0 and week 10. In the knee moment feedback group, gait pattern changes (FPA, step width and step length) from week 1 to week 6 and 1-month follow-up will be compared to explore the evolvement of the gait pattern in response to knee moment feedback intervention. The proportion of participants adequately completing the programme and the average number of training sessions completed will be reported.

All outcomes will be analysed using the intention-to-treat approach and thus we will include data from all enrolled participants, regardless of dropout. Descriptive statistics will be used to compare participants randomised into the two intervention groups and a control group with respect to baseline variables. Continuous variables will be expressed as the means and standard deviations (if normally distributed). The data normality and sphericity will be tested using Shapiro–Wilk test and Mauchly’s test respectively. Linear mixed effect (LME) models will be used to evaluate whether knee loading variables, knee pain score and WOMAC score significantly differ between the three time points (week 0, week 6 and week 10), with group as fixed effect, and participant (repeat measures) as a random effect. LME will be used for our primary objective to explore the difference between baseline assessment (week 0) and post-intervention assessment (week 6) with comparison of gait pattern group and control group, and the secondary objective to explore the gait pattern difference across all time points in knee moment group. The example LME formula for comparison between the gait pattern and control group within MATLAB is stated as below:

$${\text{glme}}\_\mathrm{KAM }=\mathrm{ fitglme}({\text{data}},\mathrm{ ^{\prime}}{\text{KAM}}1 \sim \mathrm{ Group }+\mathrm{ timepoint }+\mathrm{ timepoint}*\mathrm{Group }+ (1|{\text{Participant}})\mathrm{^{\prime}},\mathrm{ ^{\prime}}\mathrm{FitMethod^{\prime}},\mathrm{^{\prime}}\mathrm{Laplace^{\prime}})$$

Where KAM1 is the1st peak KAM; Group is the gait pattern group and control group; Timepoints includes baseline and post-training.

Repeated measures Bland–Altman analysis will be used to test the similarity between gait pattern group and knee moment group [35].

## Discussion

### Study management and safety

The University of Bath, as Sponsor, will be responsible for monitoring and auditing the study. The supervisory team will monitor the progress of the study and conduct audits to confirm appropriate data storage. The supervisors and the lead researcher will meet at the beginning, midpoint and end of the study to monitor data and adverse events, and to make recommendations regarding safety and ethical issues.

The safety and well-being of participants will be assured throughout the study. The environment and equipment are regularly examined for safety considerations. There is a small risk of falling if participants lose their balance when performing motor tasks, but the risk of falling is considered very low and is no greater than when performing these daily activities outside the laboratory. Gait retraining sessions require changes in gait patterns, which have a very small possibility of causing discomfort or a slight amount of pain during walking. If this happens, we will adjust the target gait patterns to reduce pain as soon as possible and if pain persists then the participant will be removed from the study.

### Data management and confidentiality

The University of Bath will act as the Data Controller for this study. Only the researcher team members will have access to the data. Personal information will never be identifiable in published papers or conference presentations. Data and participants information will be collected in accordance with the confidentially NHS Code of Practice and all information will be subject to the current conditions of the Data Protection Act. Each participant will be assigned a unique project code which will be used to identify the participant anonymously. An Excel data sheet maintained on a University X: Drive (password protected) will be used to record participant information. Signed consent form will be stored in a locked cupboard and kept for 10 years to evidence the consent process.

### Post-trial care

By identifying and practicing the optimal gait pattern identified in this trial, it is hoped that there will be physical benefits such as pain reduction, reduced knee loading, improved quality of life and reduced disease progression. Participants will be encouraged to keep practicing the gait pattern after they finish the study.

### Dissemination

The results of this study will be submitted for journal publications and conference presentations, which will be exposed to researchers and clinicians in the same field. We will also promote the study outcomes to a wider audience such as physiotherapists and individuals with KOA through internet media.

## Conclusion

To the best of our knowledge, this is the first study to explore the effectiveness of gait pattern biofeedback gait retraining on knee loading and knee pain in KOA population, compared to a control group. The results of this study will also identify the number of sessions needed to identify the optimal gait pattern using knee moment feedback. The similarity of effectiveness between gait pattern and knee moment feedback will be assessed as exploratory objective. The results can provide guidance of clinical implementation and further progress the development of portable gait retraining devices for use outside of the laboratory. This study will also explore the muscle activation alterations during gait retraining sessions, which can help illuminate the potential effective mechanisms of gait retraining. Furthermore, this study aims to close the knowledge gap in the literature on the translative effect of gait retraining on other daily activities, such as stair climbing and sit-to-stand. A comprehensive and effective gait retraining programme may represent a practical and self-administered approach to slow KOA progression and improve quality of life in patients with knee osteoarthritis.

## Data Availability

Not applicable.

## Abbreviations

BMI: Body mass index
CSEOR: Centre for Sport Exercise and Osteoarthritis Research
EMG: Electromyography
FPA: Foot progression angle
KAM: Knee adduction moment
KFM: Knee flexion moment
KOA: Knee osteoarthritis
LME: Linear mixed effect
MVC: Maximum voluntary contraction
NRS: Numeric Rating Scale
PPI: Patient and Public Involvement
WOMAC: Western Ontario and McMaster Universities Arthritis Index
